# ALKBH5-mediated m^6^A demethylation of lncRNA PVT1 plays an oncogenic role in osteosarcoma

**DOI:** 10.1186/s12935-020-1105-6

**Published:** 2020-01-30

**Authors:** Shuo Chen, Liwu Zhou, Yang Wang

**Affiliations:** Department of Orthopedics, Jinling Hospital, Nanjing University School of Medicine, 305 Zhongshan East Road, Nanjing, 210004 China

**Keywords:** YTHDF2, RNA stability, RNA methylation, m^6^A modification

## Abstract

**Background:**

Osteosarcoma (OS) is one of the most common malignant bone tumors. Plasmacytoma variant translocation 1 (PVT1) is a well-known oncogenic long noncoding RNA (lncRNA). However, to date, the regulatory mechanism of PVT1 upregulation in OS remains unknown.

**Methods:**

qRT-PCR was carried out to test the expression level of PVT1 and ALKBH5. RNA immunoprecipitation (RIP) and RNA pull-down assays were performed to detect the interaction of PVT1 with ALKBH5 and YTHDF2. Methylated RNA immune-precipitation (MeRIP) was used to examine the *N*^6^-methyladenosine (m^6^A) modification of PVT1 transcript.

**Results:**

In this study, we found that PVT1 expression was upregulated in OS tissues and cells and significantly related with clinical stage, tumor size, and prognosis of patients with OS. Further investigation revealed that *N*^6^-methyladenosine (m^6^A) demethylase ALKBH5 could associate with PVT1 and suppress its degradation. ALKBH5 decreased the m^6^A modification of PVT1, thus inhibiting the binding of reader protein YTHDF2 in PVT1. Functionally, ALKBH5-mediated PVT1 upregulation promoted the OS cell proliferation in vitro and tumor growth in vivo.

**Conclusions:**

Our study suggests that ALKBH5-mediated m^6^A modification of PVT1 contributes to OS tumorigenesis.

## Background

Osteosarcoma (OS) is one of the most common malignant bone tumors and mainly occurs in children and adolescents [1]. The surgical resection combined chemotherapy is the main treatment for patients with OS. After standardized treatment, OS patients without distant metastasis usually had a relatively good prognosis [2]. Unfortunately, the 5-year survival rate of OS patients with distant metastasis is only about 5–20% [3]. Thus, a better understanding of underlying mechanism promoting OS progression are urgently needed, which may be helpful for developing more effective treatment strategies for patients.

The *N*^6^-methyladenosine (m^6^A), a dynamic and reversible modification, is the most abundant in eukaryotic RNAs [4]. The m^6^A modification is regulated by some enzymes. Methyltransferase-like 3 (METTL3), METTL14, and Wilms tumor 1-associated protein (WTAP) act as m^6^A methyltransferases (“writers”). m^6^A is removed by two demethylases (“eraser”) fat mass and obesity-associated protein (FTO) and α-ketoglutarate-dependent dioxygenase AlkB homolog 5 (ALKBH5). Moreover, m^6^A-binding proteins, including YTHDF1, YTHDF2, YTHDF3 and YTHDC1, have been identified to be the “readers” of m^6^A modification and regulate pre-mRNA processing, translation, and degradation [5]. Recently, abnormal m^6^A modification is revealed to be associated with tumorigenesis, proliferation, differentiation, invasion and distant metastasis, and function as oncogenes or tumor suppressor in human cancers [6].

Long noncoding RNAs (lncRNAs) are defined as transcripts larger than 200 nt without protein coding capacity. lncRNAs have been found to act as crucial regulators in various aspects of cell biological behaviors, such as cell proliferation, apoptosis, invasion, differentiation and autophagy [7]. Mechanically, lncRNAs interact with other molecules and subsequently participate in the regulation of histone modification, gene transcription, RNA stability, RNA splicing, microRNA activity, transcriptional or translational modification [8]. Recently, lncRNAs have been reported to regulate m^6^A modification. lncRNA FOXM1-AS promotes the interaction of m^6^A demethylase ALKBH5 with FOXM1 nascent transcripts, which enhances tumorigenicity of glioblastoma stem-like cells [9]. In cervical cancer, lncRNA GAS5-AS1 recruits ALKBH5 to tumor suppressor GAS5, thus decreasing GAS5 m^6^A methylation and stabilizing it [10]. Additionally, abnormal m^6^A modification contributes to the dysregulation of lncRNAs in human cancers. For example, m^6^A was highly enriched within lncRNA FAM225A and enhanced its RNA stability, resulting in nasopharyngeal carcinogenesis and metastasis [11]. m^6^A methylation was involved in the upregulation of lncRNA RP11 by increasing its nuclear accumulation in colorectal cancer cells [12]. However, to date, the relationship between lncRNAs-mediated m^6^A methylation and OS progression remains unclear.

Plasmacytoma Variant Translocation 1 (PVT1) is a well-known oncogenic lncRNA. PVT1 is found to be be upregulated in several type of human cancers, including bladder cancer, hepatocellular carcinoma, cervical cancer, gastric cancer, lung cancer, prostate cancer and OS [13–15]. It has been revealed that PVT1 promotes cancer initiation and progression via acting as competing endogenous RNA (ceRNA) or activating STAT3 signaling or KAT2A acetyltransferase or interacting with MYC [15–17]. But little is known about regulatory mechanism of PVT1 overexpression in cancers. Zhao et al. identified PVT1 as a STAT3-responsive lncRNA, and found that STAT3 could occupy the *PVT1* promoter to activate its transcription [17]. Additionally, transcription factor RUNX2, SOX2, FOXM1 and YY1 is capable to upregulate PVT1 expression [18–21]. However, whether the m^6^A modification is responsible for the upregulation of PVT1 expression in OS is still unrevealed.

Here, we found that PVT1 was a valuable prognostic predictor of patients with OS and revealed a novel regulatory mechanism of PVT1 upregulation. ALKBH5-mediated m^6^A demethylation facilitated the stability of PVT1, which promoted OS growth. ALKBH5-PVT1 may appear to be a promising target for OS therapy.

## Materials and methods

### Tissue samples

70 pairs of OS and adjacent normal tissues were collected from OS patients who underwent surgical resection at Jinling Hospital from January 2013 to December 2018. None of the patients received chemotherapy or radiotherapy before surgery. Two experienced pathologists diagnosed and defined the tumor stage independently. All the samples were snap-frozen in liquid nitrogen and then stored at − 80 °C until used. All patients provided written informed consent. This research was approved by the Ethics Committee of Jinling Hospital and carried out in accordance with in accordance with the World Medical Association Declaration of Helsinki.

### Cell culture and transfection

Six OS cell lines (LM7, SaOS2, HOS, U2OS, MG63 and 143B cells) and a normal osteoblast cell line (Nhost) were obtained from the Institute of Biochemistry and Cell Biology of the Chinese Academy of Sciences (Shanghai, China) and cultured in Dulbecco’s modified Eagle’s medium (DMEM) (Invitrogen) medium supplemented with 10% fetal bovine serum (FBS) at 37 °C in an atmosphere containing 5% CO_2_. pLKO.1 plasmid expressing scramble or YTHDF2 or PVT1 shRNAs were constructed and purchased from GenePharma Company. Scramble or YTHDF2 or PVT1 shRNAs were transfected into OS cells by using Lipofectamine 2000 (Invitrogen, USA). 48 h after transfection, the cells were used for further detection.

### Knockdown and overexpression of ALKBH5

Lentivirus expressing scramble or ALKBH5 shRNAs was purchased from GenePharma Company. In the case of knockdown experiments, cells were infected these lentiviral particles and selected with 3 μg/ml puromycin. In the case of overexpression experiments, cells were infected with lentiviral particles expressing empty vector control or ALKBH5 (GenePharma Company) and selected with 3 μg/ml puromycin.

### RNA isolation and qRT-PCR

Total RNA was isolated using RNeasy Mini Kit (Qiagen) and reversely transcribed using PrimeScript™ RT reagent Kit according to the instruction. The relative expression of indicated genes was quantified by qRT-PCR using SYBR Premix ExTaq kit and was normalized to the expression of GAPDH. Relative changes in expression were calculated using the 2^−ΔΔCt^ method. The primers for qRT-PCR were shown as follow: GAPDH, forward 5′-GGTGTGAACCATGAGAAGTATGA-3′ and reverse 5′-GAGTCCTTCCACGATACCAAAG-3′; PVT1, forward 5′-GAATAACGGGCTCCCAGATT-3′ and reverse 5′-CCTGAGTCTCAAGATGCAGTAG-3′; ALKBH5, forward 5′-GCTTCAGGGTATGGGAGTTG-3′ and reverse 5′-TTCCAGGATCTGAGTGGATAGA -3′.

### Western blot

Cells were ruptured with RIPA buffer (Beyotime) containing cocktail inhibitor (Roche). Cell lysates were resolved by SDS-PAGE and transferred onto PVDF membranes (Millipore). The membranes were blocked and then incubated with primary antibodies overnight at 4 °C. Specific antibodies used are listed below: METTL3 (Cell Signaling Technology), YTHDF2 (Cell Signaling Technology) and GAPDH (Proteintech). Subsequently, the membranes were incubated with corresponding secondary antibodies and detected by ECL Western Blotting Substrate (Thermo).

### Cell proliferation detection

Cell proliferation was determined by Cell Counting Kit 8 (CCK-8) and colony formation assays. For CCK-8 assay, cells were seeded in 96-well plates (2000 cells per well). At the indicated time points, 10 μL CCK-8 reagent (Dojindo) was added and cells incubated for another 1 h at 37 °C. The optical density at 450 nm was measured. For colony formation assay, 2000 cells were plated in 6-well plates. After 2 weeks, cells were fixed with 10% paraformaldehyde and stained with 0.2% crystal violet.

### In vivo animal study

1 × 10^7^ indicated OS cells were subcutaneously injected into 4-week-old male athymic nude mice. Tumor volume measured at the indicated times. After 35 days, the mice were sacrificed, and the tumor weight was measured. These animal experiments were carried out according to the NIH Guide for the Care and Use of Laboratory Animals.

### RNA immunoprecipitation assay (RIP)

RIP assay was performed using a Magna RNA-binding protein immunoprecipitation kit (Millipore) according to its instruction. 3 μg METTL3, METTL14, WTAP, FTO, ALKBH5, YTHDF2, YTHDF3, YTHDC2 and IgG control antibodies were used for RIP assay. Co-precipitated RNAs were then detected by qRT-PCR.

### RNA pull-down assay

RNA pull-down was performed using Pierce™ Magnetic RNA–protein pull-down kit (Thermo) according to its instruction. The RNA–protein complex was analyzed by western blot.

### Methylated RNA immune-precipitation (MeRIP) assay

Total RNA was isolated from OS cells. m^6^A antibody (Abcam) and Magna methylated RNA immune-precipitation (MeRIP) m^6^A Kit (Merck Millipore) was used to immunoprecipitate chemically fragmented RNA (~ 100 nucleotides) according to its instruction. Enrichment of m^6^A containing RNA was then tested via qRT-PCR.

### Luciferase reporter assay

Wild-type or m^6^A consensus sequence mutant PVT1 was cloned into luciferase reporter pmirGLO. Stable OS cells were transfected with these plasmids. After 48 h, the luciferase activity was tested using Dual-Luciferase^®^ Reporter Assay System.

### Statistical analysis

Statistical analysis was performed using GraphPad Prism 7.0. Two-tailed Student’s t test, ANOVA test, Chi square test, Pearson correlation and Kaplan–Meier analysis were used as appropriate. A p value of less than 0.05 was considered significant.

## Results

### PVT1 is a valuable prognostic predictor of patients with OS

**Fig. 1:**
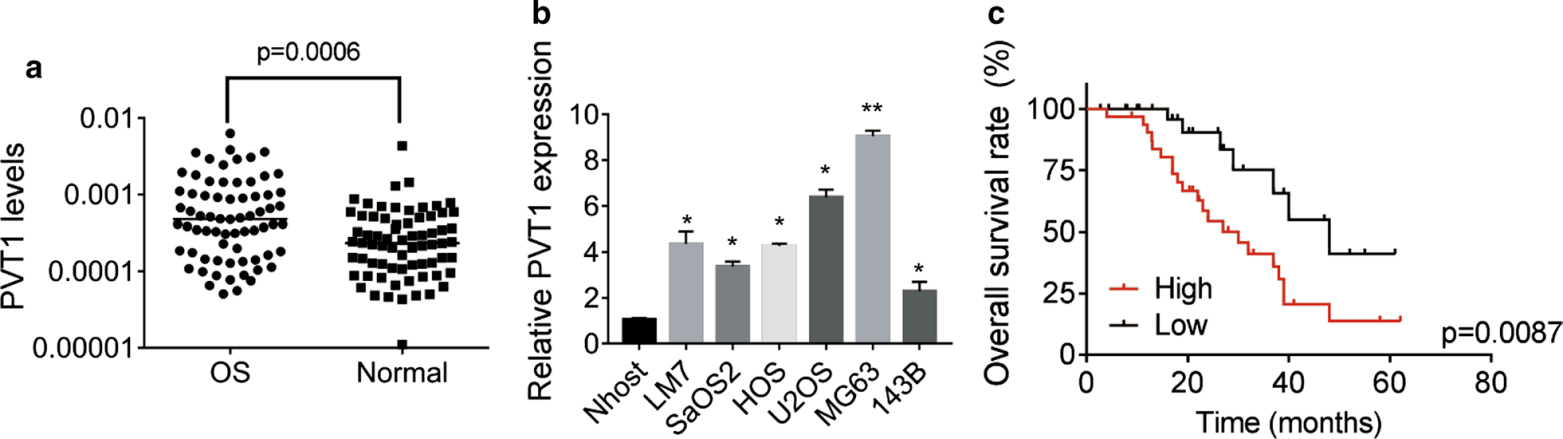
Overexpression of PVT1 in OS tissues predicts poor prognosis of patients. **a** qRT-PCR analysis of PVT1 levels in 70 pairs of OS and adjacent normal tissues. **b** qRT-PCR analysis of PVT1 levels in six OS cell lines and a normal osteoblast cell line Nhost. **c** Patients were divided into two groups based on the median value of PVT1 expression in OS tissues. Kaplan–Meier and log-rank test was used to evaluate the correlation between PVT1 expression and prognosis of patients with OS. *p < 0.05, **p < 0.01

Firstly, we carried out qRT-PCR assay to detect the expression of PVT1 in 70 pairs of OS and adjacent normal tissues. The results demonstrated a significant increase of PVT1 expression in OS tissues compared to normal tissues (Fig. 1a). It is also observed that PVT1 was much higher in OS cell lines than that in a normal osteoblast cell line Nhost (Fig. 1b). To analyze the relationship between PVT1 expression and clinicopathological features, patients were divided into two groups based on the median value of PVT1 expression in OS tissues. We found that PVT1 expression was significantly correlated with clinical stage and tumor size (Table 1). Additionally, survival analysis via Kaplan–Meier and log-rank test showed that patients with high PVT1 expression had a worse prognosis than those with low PVT1 expression did (Fig. 1c). There data suggested that upregulation of PVT1 was associated with OS progression and poor clinical outcome.

**Table 1:** Correlation analysis between lncRNA PVT1 expression and clinicopathological features in patients with OS

Parameters	PVT1 expression		p value
	Low (n = 35)	High (n = 35)	
Gender			
Male	16	18	0.632
Female	19	17	
Age			
>20	15	16	0.810
≤ 20	20	19	
Location			
Femur/Tibia	19	17	0.632
Elsewhere	16	18	
Tumor size(cm)			
≤ 5	20	8	0.003
>5	15	27	
Tumor stage			
I + IIA	21	10	0.008
IIB/III	14	25	
Metastasis			
Yes	23	20	0.461
No	12	15	

### ALKBH5 associates with PVT1 and upregulates its expression

**Fig. 2:**
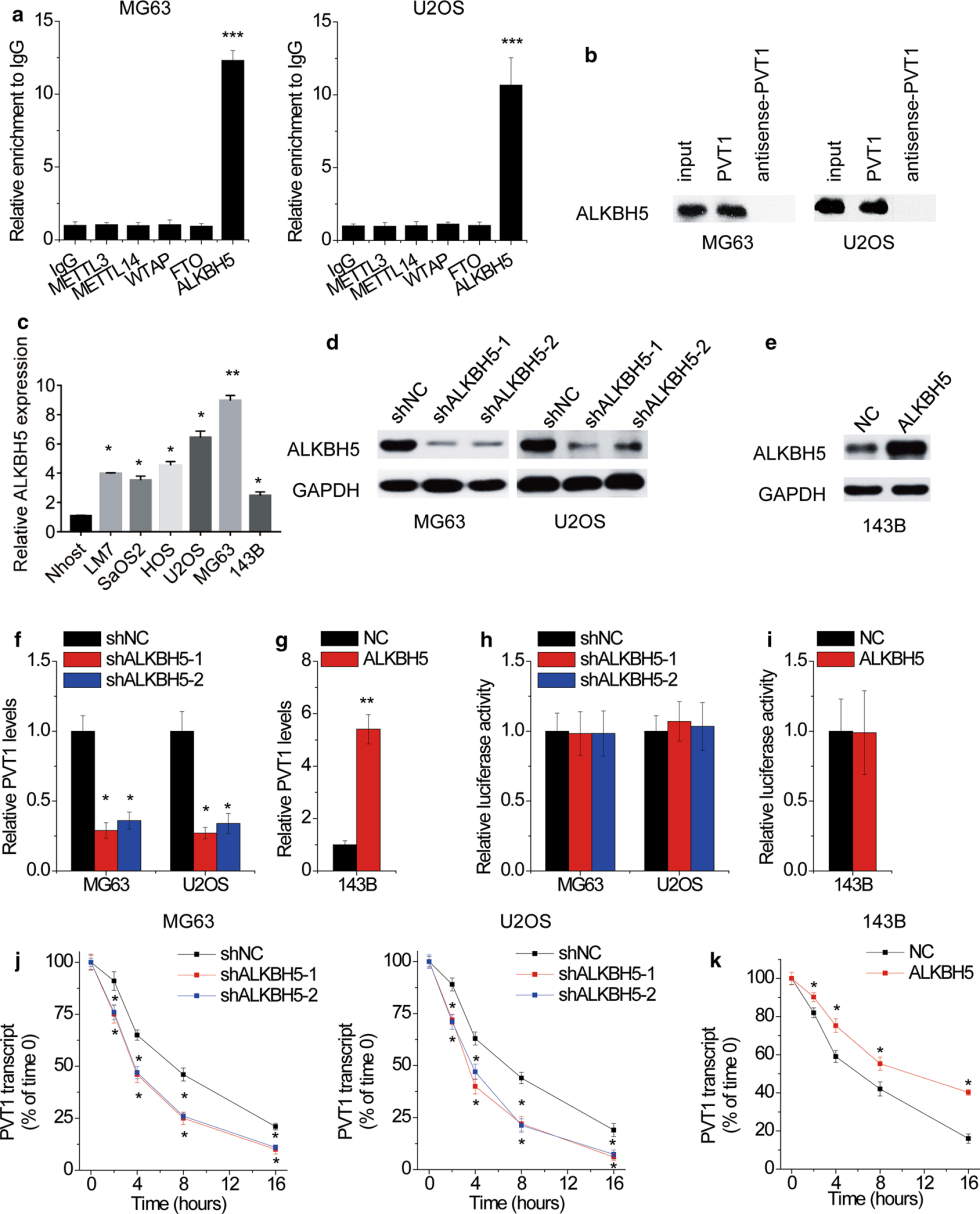
ALKBH5 associates with PVT1 and inhibits its stability. **a** The RIP assays using METTL3, METTL14, WTAP, FTO and ALKBH5 antibodies were carried out. **b** MG63 and U2OS cell lysates were incubated with biotin-labeled PVT1 or antisense PVT1; after pull-down, ALKBH5 was detected by western blot. **c** qRT-PCR analysis of ALKBH5 mRNA levels in six OS cell lines and a normal osteoblast cell line Nhost. **d** MG63 and U2OS cells were infected with lentivirus expressing scramble control shRNA (shNC) or ALKBH5 shRNAs (shALKBH5). The knockdown efficacy was validated by qRT-PCR. **e** 143B cells were infected with lentivirus expressing empty vector control (NC) or ALKBH5. The overexpression efficacy was validated by qRT-PCR. **f** The qRT-PCR analysis of PVT1 levels in control and ALKBH5-silenced MG63 and U2OS cells. **g** The qRT-PCR analysis of PVT1 levels in control and ALKBH5-overexpressed 143B cells. **h** The luciferase reporter containing *PVT1* promoter was transfected into MG63 and U2OS cells with ALKBH5 knockdown. The luciferase activity was measured. **i** The luciferase reporter containing *PVT1* promoter was transfected into 143B cells with ALKBH5 overexpression. The luciferase activity was measured. **j** Control and ALKBH5-silenced MG63 and U2OS cells were treated with α-amanitin(50 mM) to block new RNA synthesis. The stability of PVT1 over time was measured by qRT-PCR relative to time 0. **k** Control and ALKBH5-overexpressed 143B cells were treated with α-amanitin(50 mM) to block new RNA synthesis. The stability of PVT1 over time was measured by qRT-PCR relative to time 0. *p < 0.05, **p < 0.01, ***p < 0.001

We then investigated the regulatory mechanism of PVT1 upregulation in OS. To investigate whether m^6^A modification was responsible for the upregulation of PVT1, we carried out RIP assays using the major m^6^A modifying enzymes (METTL3, METTL14, WTAP, FTO and ALKBH5) antibodies. Notably, PVT1 could be significantly enriched by ALKBH5, compared to IgG, METTL3, METTL14, WTAP and FTO (Fig. 2a). The results of RNA pull-down assay further validated the interaction between PVT1 and ALKBH5 (Fig. 2b). Additionally, we detected the ALKBH5 expression in OS cell lines and a normal osteoblast cell line and found that ALKBH5 expression was overexpressed in OS cell lines and showed a positive correlation with PVT1 (Fig. 2c), indicating that ALKBH5 might affect PVT1 expression.

Given the endogenous ALKBH5 expression, we silenced ALKBH5 expression in MG63 and U2OS cells (Fig. 2d), and stably overexpressed ALKBH5 expression in 143B cells (Fig. 2e). It was demonstrated that silence of ALKBH5 resulted in an obviously decrease in PVT1 expression (Fig. 2f), where ectopic expression of ALKHB5 promoted PVT1 expression (Fig. 2g). We then evaluated the possible mechanisms involved in the ALKBH5-mediated PVT1 upregulation. Luciferase reporter plasmid containing *PVT1* promoter was constructed and transfected into above stable OS cells. However, ALKHB5 did not affect the activity of *PVT1* promoter (Fig. 2h, i), implying that ALKBH5 could not active PVT1 transcription. By treating OS cells with α-amanitin to block RNA synthesis, our data revealed that depletion of ALKBH5 expression significantly decreased the half-life of PVT1 in MG63 and U2OS cells (Fig. 2j). Conversely, the degradation of PVT1 was inhibited by ALKBH5 upregulation in 143B cells (Fig. 2k). Taken together, these data indicated that ALKBH5 interacted with PVT1 and increased its stability.

### ALKBH5 demethylates PVT1 transcripts

**Fig. 3:**
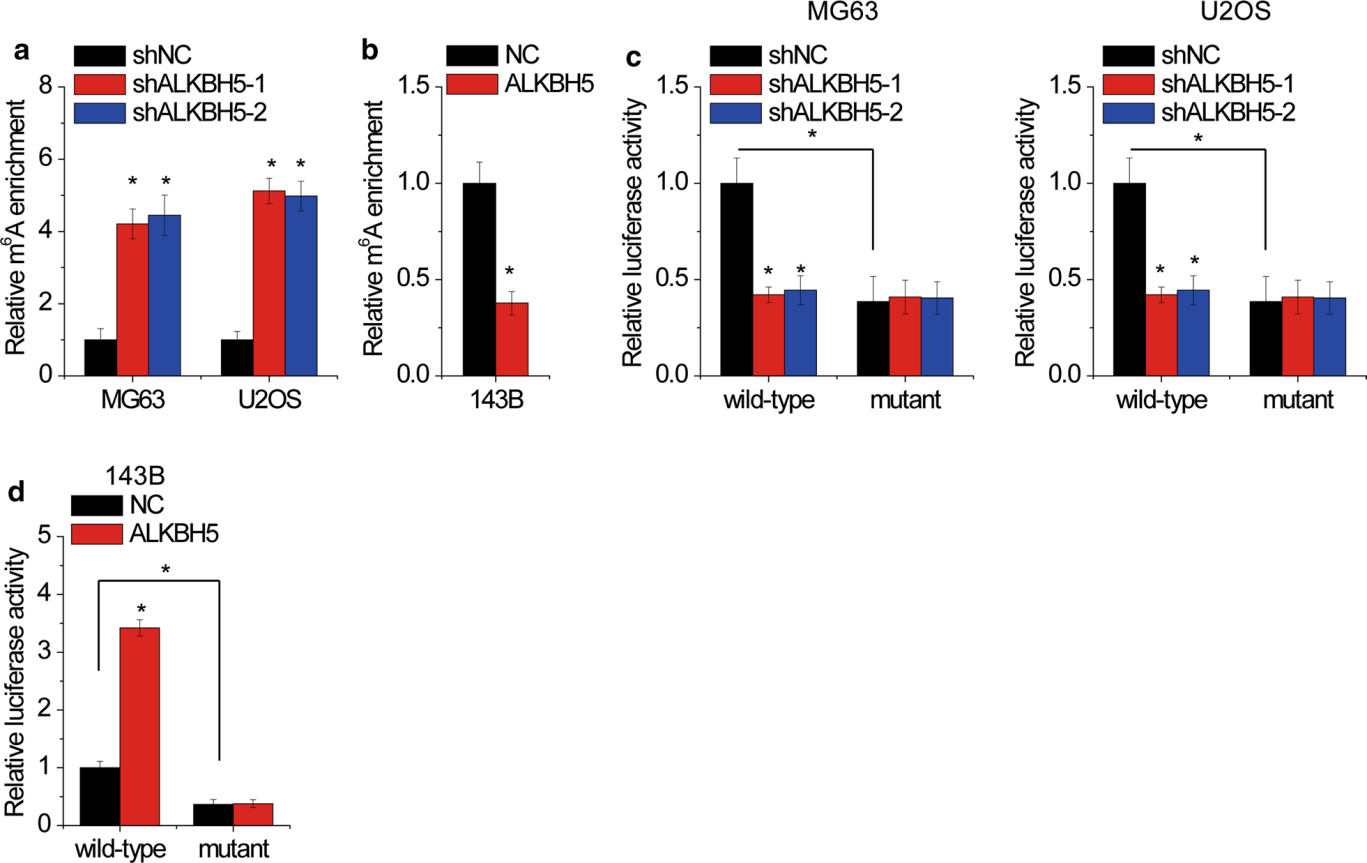
ALKBH5 demethylates PVT1 transcripts. **a** MeRIP assay was performed to detect the effect of ALKBH5 knockdown on m^6^A modification of PVT1 in MG63 and H2OS cells. **b** MeRIP assay was performed to detect the effect of ALKBH5 overexpression on m^6^A modification of PVT1 in 143B cells. **c** Luciferase reporter containing wild-type or m^6^A consensus sequence mutant PVT1 was transfected into MG63 and U2OS cells with ALKBH5 knockdown. The luciferase activity was then detected. **d** Luciferase reporter containing wild-type or m^6^A consensus sequence mutant PVT1 was transfected into 143B cells with ALKBH5 overexpression. The luciferase activity was then detected. *p < 0.05

As ALKBH5 is a RNA demethylase, ALKHB5 may suppress the m^6^A modification of PVT1. To validate this, methylated RNA immune-precipitation (MeRIP) assay was performed. It was identified that PVT1 transcripts could be significantly enriched by m^6^A-specific antibody in OS cells. Knockdown of ALKBH5 increased the m^6^A level of PVT1 in MG63 and U2OS cells (Fig. 3a), whereas ALKBH5 overexpression impaired m^6^A methylation of PVT1 in 143B cells (Fig. 3b). m^6^A modification tends to occur at a subset of RRACH motifs (R = G or A; H = A, C or U). We identified 19 RRACH motifs within PVT1 (Additional file 1: Figure S1) and then mutated these motifs (5′-RRACU-3′ to 5′-RRUCU-3′) to abolish the m^6^A modification. The results of luciferase reporter assay demonstrated that mutation of m^6^A consensus sequences suppressed the activity of PVT1-fused reporter in OS cells (Fig. 3c, d). Downregulation of ALKBH5 obviously decreased the luciferase activity of PVT1-fused reporter in MG63 and U2OS cells (Fig. 3c), whereas silence of ALKBH5 reversed this effect in 143B cells (Fig. 3d). However, ALKBH5 did not affect the luciferase activity of mutant PVT1-fused reporter. Taken together, these data indicated that PVT1 expression was regulated ALKBH5-mediated m^6^A demethylation.

### YTHDF2 is crucial for ALKBH5-mediated suppression of PVT1 degradation

**Fig. 4:**
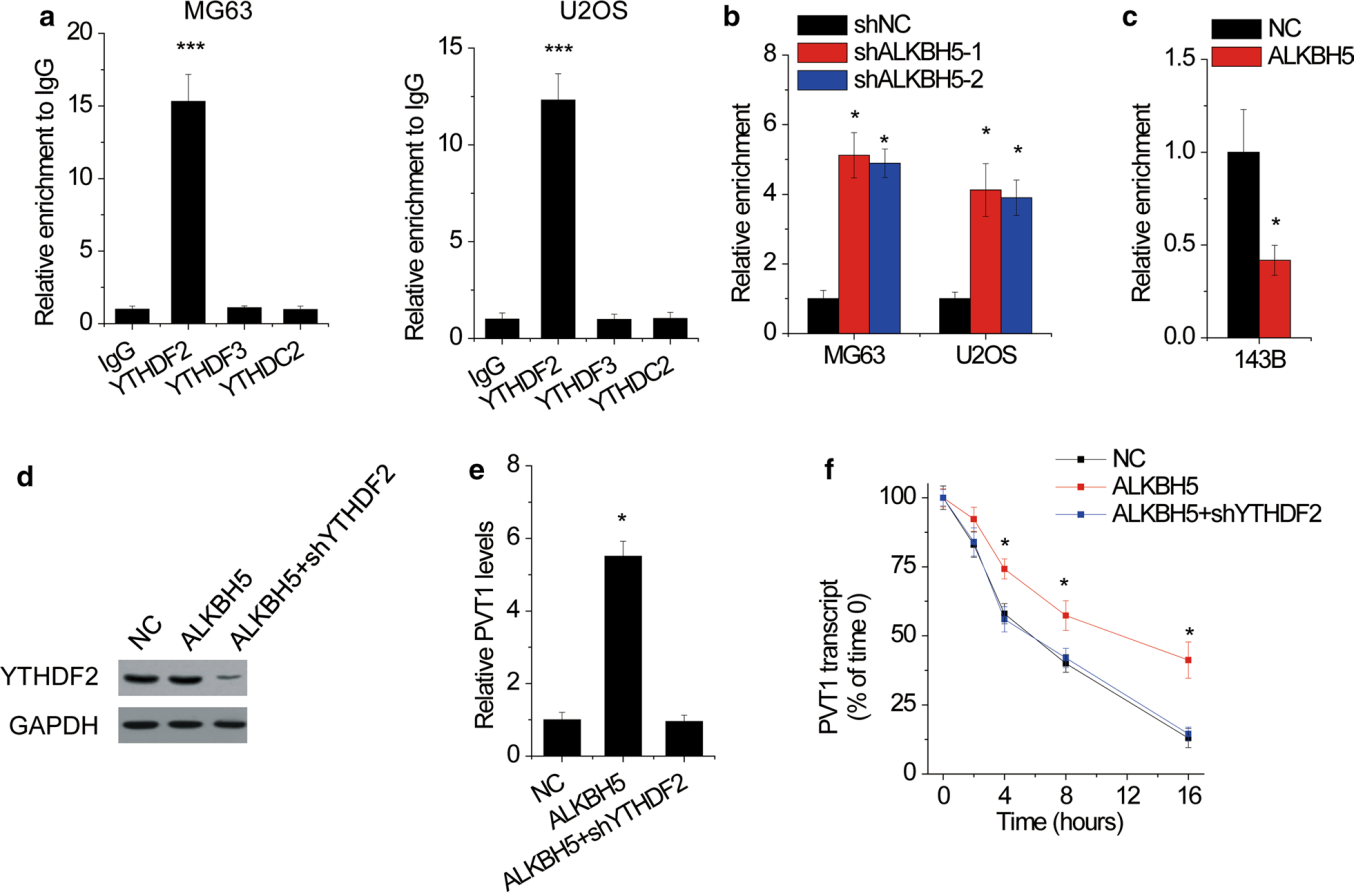
YTHDF2 is crucial for ALKBH5-mediated suppression of PVT1 degradation. **a** The RIP assays using YTHDF2, YTHDF3 and YTHDC2 antibodies were carried out in MG63 and H2OS cells. **b** The effect of ALKBH5 knockdown on the interaction between YTHDF2 and PVT1 was measured using anti-YTHDF2 RIP assay in MG63 and H2OS cells. **c** The effect of ALKBH5 overexpression on the interaction between YTHDF2 and PVT1 was measured using anti-YTHDF2 RIP assay in 143B cells. **d** The YTHDF2 expression was knocked down in ALKBH5-overexpressed 143B cells. **e** The PVT1 level in ALKBH5-overexpressed 143B cells transfected with YTHDF2 shRNA. **f** The stability of PVT1 over time in ALKBH5-overexpressed 143B cells transfected with YTHDF2 shRNA was measured by qRT-PCR relative to time 0. *p < 0.05, ***p < 0.001

Reader protein YTHDF2, YTHDF3 and YTHDC2 was identified to specially bind m^6^A-modified RNAs and lead to degradation of these RNAs [22]. To examine which reader protein was responsible for ALKBH5-mediated PVT1 upregulation, we conducted RIP assays using YTHDF2, YTHDF3 and YTHDC2 antibodies. The results identified that only YTHDF2 could significantly associate with PVT1 (Fig. 4a). Additionally, the interaction between PVT1 and YTHDF2 was strengthened after silencing ALKBH5 expression in MG63 and U2OS cells (Fig. 4b), while suppressed by ALKBH5 overexpression in 143B cells (Fig. 4c).

To detect YTHDF2 was involved in ALKBH5-mediated PVT1 upregulation, the YTHDF2 was knocked down in ALKBH5-overexpressed 143B cells (Fig. 4d). It was demonstrated that knockdown of YTHDF2 attenuated the ALKBH5-induced PVT1 upregulation (Fig. 4e). Additionally, the half-life of PVT1 in 143B cells with ALKBH5 upregulation was reversed after deletion of YTHDF2 expression (Fig. 4f). These data suggested that YTHDF2 was critical for ALKBH5-mediated PVT1 stability.

### ALKBH5 is upregulated in OS and correlated with PVT1 expression

**Fig. 5:**
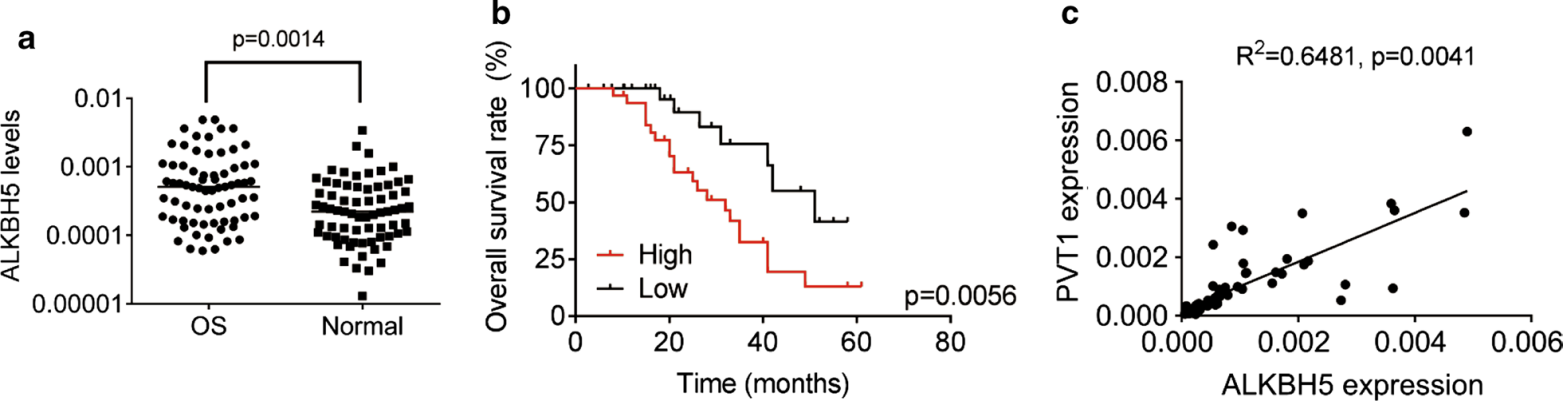
ALKBH5 is upregulated in OS and correlated with PVT1 expression. **a** The qRT-PCR analysis of ALKBH5 mRNA levels in 70 pairs of OS and adjacent normal tissues. **b** Patients were divided into two groups based on the median value of ALKBH5 expression in OS tissues. Kaplan–Meier and log-rank test was used to evaluate the correlation between PVT1 expression and prognosis of patients with OS. **c** Correlation between ALKBH5 mRNA and PVT1 expression in OS samples are shown (by Pearson correlation test)

We then determined the expression levels of ALKBH5 in OS tissues using qRT-PCR. The results showed that ALKBH5 mRNA levels were obviously upregulated in OS tissues compared to normal tissues (Fig. 5a). Survival analysis revealed that overexpression of ALKBH5 was associated with poorer prognosis of patients with OS (Fig. 5b). Moreover, ALKBH5 mRNA level was positively correlated with PVT1 transcript level (Fig. 5c). These results indicated that ALKBH5 and PVT1 could be potential prognostic markers for OS patients.

### ALKBH5 promotes OS growth partially through PVT1

**Fig. 6:**
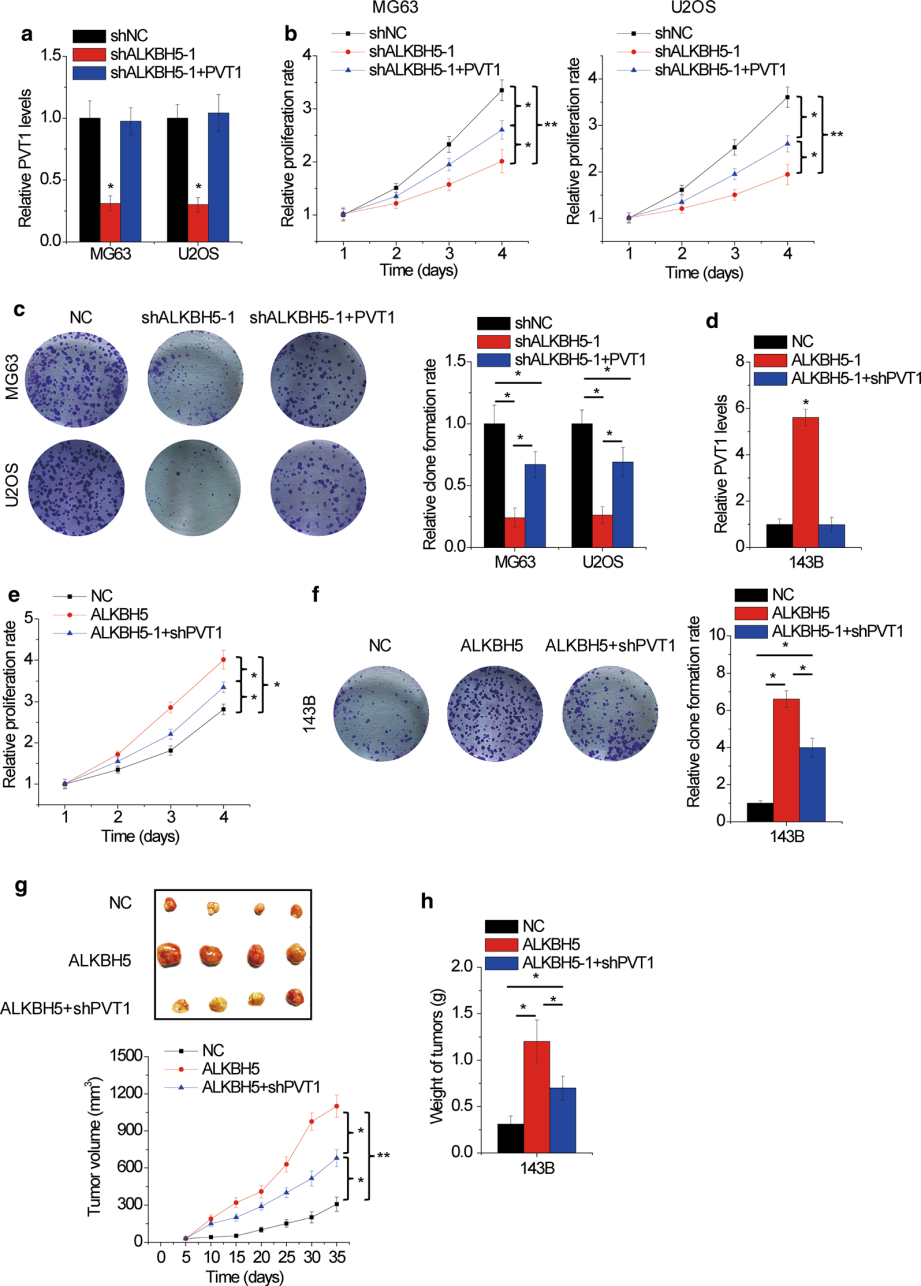
ALKBH5 promotes OS growth partially through PVT1. **a** PVT1 was overexpressed in MG63 and H2OS cells with ALKBH5 knockdown. **b** Restoration of PVT1 expression partly rescued the suppression of MG63 and H2OS cell proliferation mediated by ALKBH5 knockdown as evidenced by CCK-8 assay. **c** Restoration of PVT1 expression partly rescued the suppression of MG63 and H2OS cell proliferation mediated by ALKBH5 knockdown as evidenced by colony formation assay. **d** PVT1 was silenced in 143B cells with ALKBH5 overexpression. **e** PVT1 knockdown partly attenuated the promotion of 143B cell proliferation mediated by ALKBH5 overexpression as evidenced by CCK-8 assay. **f** PVT1 knockdown partly attenuated the promotion of 143B cell proliferation mediated by ALKBH5 overexpression as evidenced by colony formation assay. **g** Indicated stable 143B cells were subcutaneously injected into nude mice. The tumor growth curve was measured. **h** The tumor weight from (**g**) was measured. *p < 0.05, **p < 0.01

Finally, we explored the functional significance of ALKBH5-PVT1 axis in OS growth in vitro and in vivo. The results of CCK-8 and colony formation assays showed that ALKBH5 knockdown significantly attenuated OS cell proliferation, while restoration of PVT1 expression partly rescued this suppressive effect in MG63 and H2OS cells (Fig. 6a–c). In contrast, proliferative ability was obviously enhanced by ALKBH5 overexpression, which was partially reversed by PVT1 silence in 143B cells (Fig. 6d–f). We then verify the functional role of ALKBH5-PVT1 axis in vivo. Compared with the control groups, mice with ALKBH5 overexpression in 143B cells exhibited a significant promotion of tumor growth in vivo. PVT1 knockdown was capable to partly counteract the protumorigenic activities of ALKBH5 (Fig. 6g, h). These results emphasized the importance of ALKBH5-PVT1 regulation in OS tumorigenesis.

## Discussion

Previous studies demonstrated a significance increase and the prognostic value of PVT1 expression in OS patients [15, 23]. Mechanistically, PVT1 targets miR-152 to enhance chemoresistance of OS to gemcitabine through activating c-MET/PI3K/AKT pathway [15]. PVT1 enhances OS cell proliferation, migration and invasion via suppressing miR-195 and enhancing the expression of its targets, such as BCL2, CCND1, and FASN [14]. Moreover, PVT1 suppresses miR-497-mediated HK2 inhibition, thus positively regulating glycolysis and OS progression [23]. Consistent with these reports, our findings also identified that PVT1 was upregulated in OS tissues and markedly correlated with clinical stage, tumor size, and overall survival of patients. Recently, the significance of plasmacytoma PVT1 expression in OS patients was determined. The results showed that high expression of PVT1 predicted low overall survival rate of OS patients and positively correlated with tumor size, TNM stage lymph node metastasis, and distant metastasis [24]. Above all, these findings highlighted the role of PVT1 as a biomarker for the prognosis of patients with OS.

The mechanisms of oncogenic roles of PVT1 have been well studied. However, the dysregulation of PVT1 expression remains largely unknown, especially in OS. Here, we identified a novel regulatory mechanism of PVT1 overexpression in OS. Through performing RIP and RNA pull-down assay, we demonstrated an interaction between PVT1 and RNA demethylases ALKBH5. ALKBH5 increased PVT1 expression via inhibiting its degradation. Mechanistically, ALKBH5 reduced the m^6^A methylation level of PVT1, subsequently decreasing the binding level of PVT1 in reader protein YTHDF2 which could increase turnover of m^6^A-modified mRNA by promoting co-localization with decay factors [22]. ALKBH5 was identified as the main demethyltransferase critical for the m^6^A methylation [25]. It has been reported that ALKBH5 functioned as an oncogene. ALKBH5 stabilizes FOXM1 mRNA to promote tumorigenesis in glioblastoma [9]. ALKBH5 suppresses NANOG and KLF4 degradation, which promotes breast cancer progression [26]. Herein, we also revealed that ALKBH5 was overexpressed in OS tissues and negatively associated with clinical outcomes of patients. Additionally, ALKBH5 promoted OS cell proliferation in vitro, and facilitated tumor growth in vivo. Nevertheless, depletion of PVT1 could not abolish the promotive effects of ALKBH5 on OS, implying that ALKBH5-mediated m^6^A demethylation of other transcripts may be involved in OS progression.

This study gain insight into the involvement of ALKBH5-mediated m^6^A demethylation of lncRNA in regulating OS progression. Some limitations of our research should not be ignored. Above all, only 70 pairs of OS tissues and matched normal tissues were used to test the differential expression of ALKBH5 and PVT1. Whether combination of ALKBH5 and PVT1 expression could be taken as a prognostic biomarker for OS requires more sample data. Next, which RRACH motif within PVT1 is more dominant for ALKBH5-mediated PVT1 upregulation also needs further investigation.

## Conclusion

In sum, our findings revealed that ALKBH5 is responsible for PVT1 upregulation in OS. As a target of ALKBH5, PVT1 partially mediates the oncogenic role of ALKBH5 in OS growth, suggesting that ALKBH5 and PVT1 may be potential therapeutic target for OS treatment.

## Supplementary information

**Additional file 1: Figure S1.** The 19 RRACH motifs within PVT1 transcript.

## Data Availability

The datasets used during this research are available.

## Abbreviations

OS: osteosarcoma
m^6^A: *N*^6^-methyladenosine
METTL3: methyltransferase-like 3
WTAP: Wilms tumor 1-associated protein
FTO: obesity-associated protein
ALKBH5: α-ketoglutarate-dependent dioxygenase AlkB homolog 5
lncRNAs: long noncoding RNAs
PVT1: plasmacytoma variant translocation 1
ceRNA: competing endogenous RNA
MeRIP: methylated RNA immune-precipitation
