# Supplementary figures and images for: ALKBH5-mediated m6A demethylation of lncRNA PVT1 plays an oncogenic role in osteosarcoma
Source: Cancer Cell Int. 2020 Jan 30;20:34. doi: 10.1186/s12935-020-1105-6 (PMC6993345; doi:10.1186/s12935-020-1105-6)

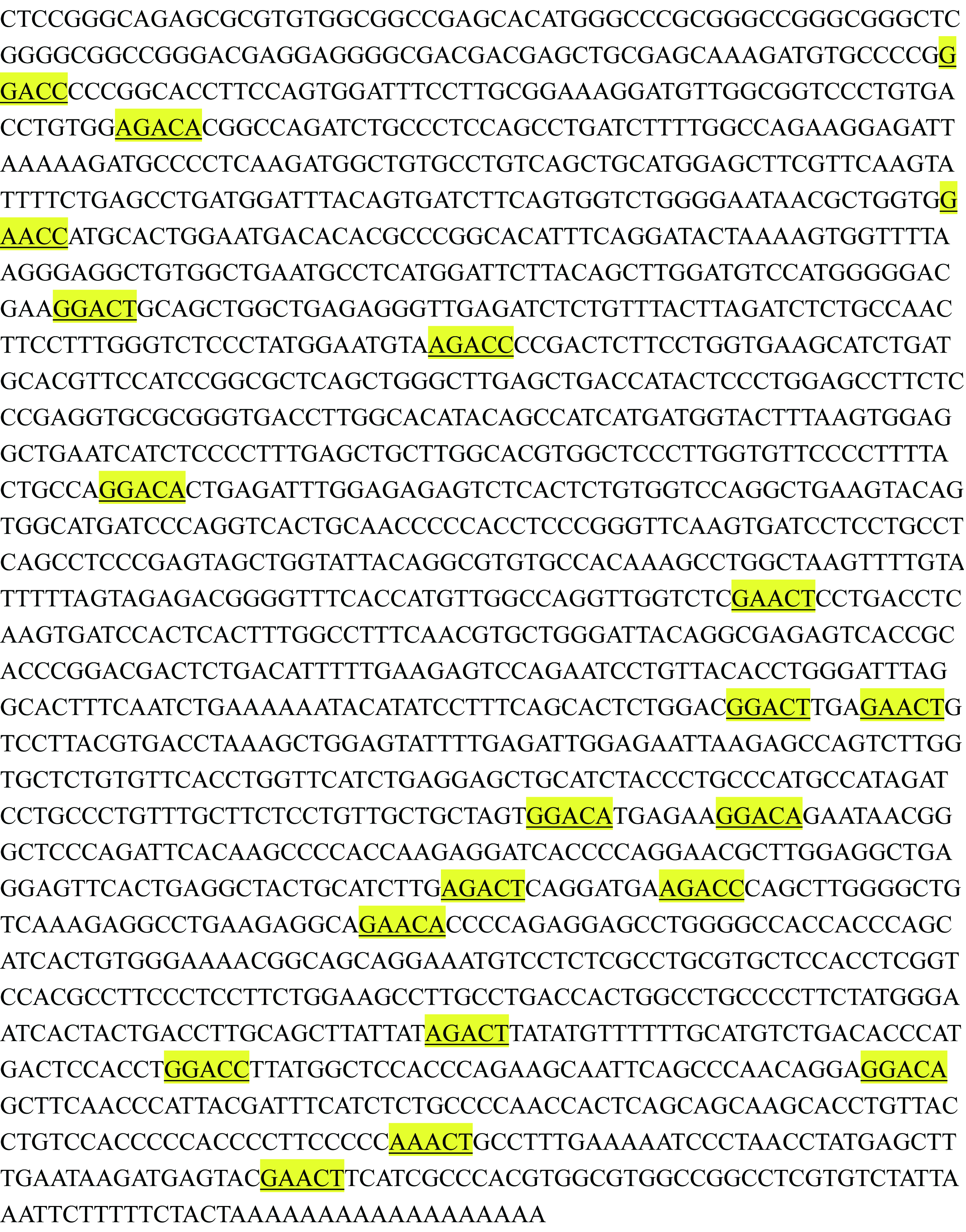

Supplement: Supplementary file 1 — Additional file 1: Figure S1. The 19 RRACH motifs within PVT1 transcript. [file 12935_2020_1105_MOESM1_ESM.tif]
